# POEMS综合征诊断与治疗中国专家共识（2025年版）

**DOI:** 10.3760/cma.j.cn121090-20241219-00578

**Published:** 2025-05

**Authors:** 中华医学会血液学分会浆细胞疾病学组 中国医师协会多发性骨髓瘤专业委员会

## Abstract

POEMS综合征是罕见浆细胞病，临床表现多样，患者易被漏诊和误诊，从而延误治疗，影响预后。POEMS综合征的诊断没有金标准，主要基于临床和实验室检查综合判断。随着自体造血干细胞移植和新药的应用，POEMS综合征的治疗和预后均获得极大改善。本共识对POEMS综合征诊断过程中存在的难点问题进行讨论，并分别对适合和不适合移植患者的治疗进行推荐。

## Abstract (en)

POEMS syndrome is a rare plasma cell disorder characterized by diverse clinical manifestations, making patients susceptible to missed diagnosis and misdiagnosis, which can delay treatment and adversely affect prognosis. There is no gold standard for diagnosing POEMS syndrome; the diagnosis primarily relies on a comprehensive assessment of clinical and laboratory findings. The application of autologous hematopoietic stem cell transplantation and novel agents has led to significant improvements in both the treatment and prognosis of POEMS syndrome. This consensus addresses the diagnostic challenges associated with POEMS syndrome and provides treatment recommendations for both transplant-eligible and transplant-ineligible patients.

POEMS综合征是一组以多发性感觉及运动性周围神经病为主要表现、常伴有多系统损害及与浆细胞异常有关的临床症候群。Scheinker在1938年首次报道本病，1980年Bardswick首次提出以五个主要临床表现的首字母命名，即多发神经病变（P，polyneuropathy）、器官肿大（O，organomegaly）、内分泌病变（E，endocrinopathy）、单克隆免疫球蛋白（M，monoclonal gammopathy）以及皮肤改变（S，skin changes）[1]。2018年5月11日，国家卫生健康委员会等5部门联合制定了《第一批罕见病目录》，POEMS综合征被收录其中。由于其罕见性、多系统受累和临床高度异质性，POEMS综合征有较高的漏诊和误诊率，从而延误患者的治疗进而影响预后[2]。为提高中国医师对这类疾病的认识水平并规范其诊治，中华医学会血液学分会浆细胞疾病学组、中国医师协会多发性骨髓瘤专业委员会组织相关专家制定了中国POEMS综合征诊治专家共识（2025年版）。

一、临床表现

POEMS综合征是一种可累及全身各器官的疾病[3]–[5]，呈慢性过程。患者初诊科室往往并不是血液科，而是就诊于神经科、消化内科、内分泌科、肾科、呼吸科、皮肤科等科室，极易被误诊。主要临床表现为多发性周围神经病变、肝脾和淋巴结肿大、内分泌改变（包括性腺、甲状腺、肾上腺、胰岛内分泌功能等）、单克隆免疫球蛋白（M蛋白）血症/单克隆性浆细胞增殖、皮肤改变（常见皮肤色素沉着、皮肤增厚、多毛症、肾小球样血管瘤、雷诺现象、白甲等）、继发淀粉样变表现、水肿/多浆膜腔积液、血小板增多、红细胞增多和血栓事件、骨骼病变（多为骨质硬化表现）。其他临床表现包括肺动脉高压、限制性肺病、神经肌肉呼吸功能受损和一氧化碳弥散能力受损，还可见布-加综合征、消瘦、乏力、多汗、顽固性腹泻等。

二、诊断、鉴别诊断和预后分层

（一）诊断所需项目

专家组推荐参照2023年梅奥中心发布的《POEMS综合征的诊断、危险分层和治疗更新》中POEMS综合征的诊断标准和推荐检查[4]，见表1。

**表1:** POEMS综合征诊断所需项目

项目	内容
病史询问和体格检查	神经系统病史的询问（如麻木、疼痛、无力）；月经史和性功能； 全身皮肤检查，寻找色素沉着、肢端发绀或雷诺现象、白甲、硬皮样改变、潮红或杵状指； 是否有男性乳房发育、淋巴结肿大、肝脾肿大、浆膜腔积液/水肿、反射松弛、观察步态； 眼底镜检查有无视乳头水肿
血液检查	一般检查：血常规、肝肾功能、电解质（包括钙离子）、凝血功能、β_2_-微球蛋白； 血M蛋白相关检查：血清免疫球蛋白定量、血清蛋白电泳、血免疫固定电泳、血清游离轻链； 特殊检查：血清VEGF、激素水平检测［包括性激素（睾酮、雌二醇、催乳素、黄体生成素、卵泡刺激素）、甲状腺激素（游离T3、游离T4、促甲状腺激素）、垂体-肾上腺轴（皮质醇、ACTH）］、空腹血糖、餐后血糖、糖化血红蛋白、MAG抗体（有条件时加做）
尿液检查	尿常规、24 h尿蛋白定量、24 h尿轻链检测、尿蛋白电泳、尿免疫固定电泳
骨髓检查	骨髓细胞学涂片+流式细胞术、骨髓活检+免疫组化；流式细胞术检查建议包括CD38、CD138、CD56、CD19、CD20、cκ轻链、cλ轻链；骨髓免疫组化检测至少包括CD38、CD138、κ轻链、λ轻链
影像学检查	骨骼CT或全身MRI或PET/CT检查有无骨病变、器官肿大、浆膜腔积液等；心电图、超声心动图（包括右心室收缩压和肺动脉压）、胸部CT、腹部B超等，必要时行肺功能检测（一氧化碳的弥散能力）
神经检查	神经肌电图、必要时神经活检；有条件的单位可评估总体神经病变限制性评估量表（ONLS）
其他	怀疑继发淀粉样变性者，需行骨髓或受累器官或腹壁皮下脂肪活检病理并行免疫组化检测κ轻链、λ轻链及刚果红染色

**注** VEGF：血管内皮生长因子；ACTH：促肾上腺皮质激素；MAG：髓鞘相关糖蛋白；MRI：磁共振成像；PET/CT：正电子发射计算机断层显像

（二）诊断

基于Dispenzieri等[6]在2007年提出的新修订的诊断标准，需满足2项强制性主要标准、1项其他主要标准和1项次要标准，国际骨髓瘤工作组（IMWG）2014多发性骨髓瘤及相关疾病诊断标准中POEMS综合征也采用该标准[7]，专家组推荐应用该诊断标准，见表2。

**表2:** POEMS综合征诊断标准

标准类型	内容
强制性主要标准（两项均需具备）	① 多发神经病变； ② 浆细胞克隆增殖（基本都是λ型）
其他主要标准（需具备1项）	① Castleman病； ② 硬化性骨病； ③ 血清血管内皮生长因子水平升高
次要标准（需具备1项）	① 脏器肿大（脾肿大、肝肿大或淋巴结肿大）； ② 水负荷增多（肢端水肿、腹水或胸水）； ③ 内分泌病变（肾上腺、甲状腺、垂体、性腺、甲状旁腺、胰腺）； ④ 皮肤改变（色素沉着、多毛症、肾小球样血管瘤、多血症、发绀、面红、指甲苍白）； ⑤ 视乳头水肿； ⑥ 血小板增多症/红细胞增多症
其他表现	杵状指、体重减轻、多汗症、肺动脉高压/限制性肺病、易栓症、腹泻、布-加综合征、维生素B12水平降低

专家组建议诊断POEMS综合征需要注意以下问题：

（1）关于M蛋白：POEMS综合征患者由于M蛋白水平很低，甚至会低于目前血清免疫固定电泳或血、尿本周蛋白电泳的最低检出水平，需要更敏感的质谱法来检测，鉴于后者未广泛应用于临床，下面四种情形可视为有M蛋白的证据：①应用免疫组化方法检测（κ/λ>4∶1或<1∶2，分别针对κ型和λ型患者，计数≥100个浆细胞）；②单克隆浆细胞（通过流式细胞术证实）；③血清游离轻链比值异常；④病灶活检证实为浆细胞瘤。对于没有M蛋白的患者建议由有经验的诊治单位进一步复核，不轻易诊断不伴有M蛋白的POEMS综合征。

（2）关于周围神经炎（PN）：虽然在疾病过程中几乎所有患者均有PN表现，但在初诊时只有60％的患者有PN表现。疾病早期症状可能不典型，可通过肌电图检查早期发现，必要时行神经活检。有条件的单位可定期评估总体神经病变限制性评估量表（ONLS）。

（3）关于内分泌改变：约84％的患者有内分泌病变，性腺功能减退是男性最有诊断特征的改变，其次是甲状腺异常、糖代谢异常和肾上腺功能不全。大多数患者的四个主要内分泌轴（性腺、甲状腺、葡萄糖代谢和肾上腺）存在混合内分泌病变。当患者仅表现为糖尿病和甲状腺功能减退时，不足以作为内分泌改变的次要标准。

（4）关于血管内皮生长因子（VEGF）：VEGF在POEMS综合征的诊断、疾病活动性和疗效评价中十分重要，但需注意其检测方法、血清或血浆标本和临界值。梅奥中心应用的血浆VEGF临界值为200 pg/ml（特异性95％，灵敏度68％）[8]；北京协和医院应用的血清VEGF临界值为1 200 pg/ml（特异性90.2％，灵敏度83.7％）[9]。不同国家和医疗中心报道的VEGF升高比例差异极大，且并不是所有患者VEGF均升高。目前为止，VEGF升高尚无理想的临界值，也不是诊断POEMS综合征患者的必需条件。血清VEGF水平是血浆VEGF水平的10～50倍，为提高VEGF检测的敏感性，专家组推荐直接检测血清VEGF水平。推荐血清VEGF>正常值2倍作为VEGF升高的临界值。

（三）鉴别诊断

专家组建议POEMS综合征主要与炎性脱髓鞘性多发性神经病［包括慢性炎性脱髓鞘性多发性神经病（CIDP）和吉兰-巴雷综合征（GBS）］、冷球蛋白血症、意义未明的单克隆免疫球蛋白血症（MGUS）、原发性系统性轻链型淀粉样变性、多发性骨髓瘤（包括冒烟型骨髓瘤）、孤立性浆细胞瘤、有肾脏意义的单克隆免疫球蛋白血症（MGRS）、IgM相关性周围神经病变（IgM-PN）等鉴别[4],[10]–[11]。

1. CIDP和GBS：POEMS综合征患者的早期慢性或亚急性进展性周围神经病易被误诊为CIDP和GBS，CIDP和GBS患者一般不会出现M蛋白、VEGF水平升高、骨硬化、皮肤改变等。POEMS综合征患者的周围神经病在神经电生理检查中更常见到近端神经传导速度（NCV）减慢，CIDP和GBS患者中传导阻滞更多见。对标准CIDP和GBS治疗无反应的患者应考虑POEMS综合征的可能，应进行相关检查以减少POEMS综合征的漏诊或误诊。

2. 原发性系统性轻链型淀粉样变性：周围神经病变、水肿、多浆膜腔积液、M蛋白等常和轻链型淀粉样变性混淆。轻链型淀粉样变性患者尿蛋白常以大量白蛋白尿为主要表现，受累器官或组织刚果红染色阳性，偏振显微镜下可见双折光现象，电镜下可见淀粉样物质。VEGF虽然也可见升高，但升高程度不如POEMS综合征。由于POEMS综合征轻链多为λ型，因此，继发性轻链型淀粉样变性并不少见，应仔细进行鉴别。临床工作中出现淀粉样变相关表现的患者在诊断原发性轻链型淀粉样变性前应先排除继发病因，如POEMS综合征、多发性骨髓瘤、华氏巨球蛋白血症等。

3. MGUS和冒烟型骨髓瘤：POEMS综合征早期患者容易被误诊为MGUS或冒烟型骨髓瘤，后两种疾病不推荐治疗，而POEMS综合征患者可能会陆续出现其他病变并可能进行性恶化。因此，对于有周围神经病变伴M蛋白的患者，当高度怀疑POEMS综合征时，应完善POEMS综合征的其他相关检查，一旦符合诊断标准，应立即启动针对POEMS综合征的治疗。

（四）预后分层

目前尚无分子或细胞遗传学指标预测POEMS综合征患者的预后，尚无较完善的预后分层系统。既往研究认为，高龄、白蛋白<32 g/L、杵状指、血管外容量超负荷（水肿、浆膜腔积液）、肺动脉高压、一氧化碳弥散功能受损、视乳头水肿、肾功能不全、合并Castleman病、VEGF升高等可能是POEMS综合征患者的不良预后因素。梅奥中心的结果显示，白蛋白>32 g/L、年轻和治疗后可获得血液学缓解的患者预后良好[12]。北京协和医院团队利用年龄>50岁、胸腔积液、肺动脉高压和肾小球滤过率将患者分为低危（0分）、中危（1分）和高危（2～5分），见表3[13]。专家组建议目前可采用北京协和医院团队的POEMS综合征预后分层系统。

**表3:** 北京协和医院的POEMS综合征预后分层系统[13]

危险因素	分数	危险分组	危险因素分数	10年OS率
年龄>50岁	1	低危组	0	98％
胸腔积液	1	中危组	1	75％
肺动脉高压	1	高危组	≥2	50％
eGFR<30 ml·min^−1^·（1.73 m^2^）^−1^	2			

**注** eGFR：估算肾小球滤过率；OS：总生存

五、治疗

（一）治疗目标

POEMS综合征的发病机制并不明确，患者伴低水平单克隆性浆细胞增殖，目前针对POEMS综合征治疗的报道大多采用清除浆细胞的治疗手段。临床实践证实，清除浆细胞克隆可缓解患者的临床症状，提高患者的生活质量，从而提高患者的总生存（OS）率和无进展生存（PFS）率。虽然血清VEGF是目前认为比较好的用于诊断和监测疾病活动度的指标，VEGF抑制剂如贝伐珠单抗理论上是很好的选择，但实际上仅有部分患者有反应[14]。因此VEGF抑制剂至今未被推荐为POEMS综合征患者的治疗选择。

专家组建议POEMS综合征的治疗目标为清除致病的浆细胞克隆，提高患者PFS率、OS率和生活质量，尤其对于神经病变的改善非常重要。部分患者可出现严重的心肺功能损害，需要紧急进行多专科干预，并可能需要重症监护支持。

（二）治疗需要考虑的因素

专家组建议治疗需要考虑患者的年龄和预后分层。疾病相关因素包括全身性病变累及器官损害情况；患者相关因素包括是否适合强化治疗，合并症情况如心脏、呼吸系统疾病和血栓形成风险，治疗相关不良反应和患者对治疗选择的倾向，包括给药途径（口服、皮下注射或静脉输注等）[15]。

（三）治疗方案

目前国际尚无POEMS综合征诊治指南或专家共识，POEMS综合征的治疗经历了传统药物时代、新药时代及自体造血干细胞移植（auto-HSCT）。一线应用auto-HSCT已显示出显著的临床疗效，尤其在改善神经病变方面更为突出，且可诱导持久的M蛋白反应率和VEGF反应率，从而延长患者的PFS期和OS期。

1. auto-HSCT：表4列举了国内外较大宗以auto-HSCT作为一线治疗的研究结果[16]–[18]。北京协和医院回顾性分析239例单中心接受auto-HSCT的新诊断POEMS综合征患者的长期结局，血液学完全缓解（CR_H_）率和VEGF完全缓解（CR_V_）率分别为57.3％和68.6％，90.5％的患者达到总体临床缓解。中位随访94个月，5年OS率为92.8％，至下一线治疗时间（TTNT）≥5年率为72.2％（中位TTNT为96个月）[17]。梅奥中心80例患者的研究结果显示，6年PFS率为72％，10年OS率为89％[19]。目前尚无随机对照研究比较一线治疗应用auto-HSCT与单纯化疗方案。回顾性分析显示，auto-HSCT的有效率较美法仑/地塞米松（Mel/Dex）方案更高（CR_H_率：50％对38％，*P*＝0.001；CR_V_率：66％对39％，*P*＝0.001），高风险患者从auto-HSCT治疗中的获益优于来那度胺/地塞米松（Len/Dex）方案（CR_V_率：66％对48％，*P*＝0.008）[20]。系列病例报告显示，接受以硼替佐米为基础方案治疗患者的PFS和OS劣于auto-HSCT[21]。

**表4:** auto-HSCT一线治疗POEMS综合征的疗效

研究单位	例数	TRM	5年PFS率	5年OS率	CR_H_率	CR_V_率	CR_pet_率	临床/神经系统改善率	auto-HSCT前诱导治疗率
CIBMTR[16]	331	0.9％	72.0％	91.0％	-	-	-	-	45.0％
PUMCH[17]	239	2％	72.2％	92.8％	57.3％	68.6％	-	92.6％	38.9％
EBMT[18]	127	2％	74.0％	89.0％	49.0％	-	-	-	87.0％

**注** auto-HSCT：自体造血干细胞移植；CIMBTR：国际血液与骨髓移植研究中心；PUMCH：北京协和医院；EBMT：欧洲血液和骨髓移植学会；TRM：治疗相关死亡率；PFS：无进展生存；OS：总生存；CR_H_：血液学完全缓解；CR_V_：血管内皮生长因子完全缓解；CR_pet_：PET-CT完全缓解；-：无数据

专家组建议，POEMS综合征应根据是否适合移植采取不同的治疗策略，对于危险度分层中高危患者，更推荐一线行auto-HSCT。适合移植的患者建议根据IMWG老年人身心健康评估（GA）进行评分，并参考《中国多发性骨髓瘤自体造血干细胞移植指南（2021年版）》中“移植患者的筛选和要求”[22]。

（1）移植前诱导治疗：由于POEMS综合征患者浆细胞负荷很低，移植前是否需要诱导化疗曾有争议，但研究证实移植前诱导治疗可使患者获益，auto-HSCT前达到CR_V_/CR_H_与5年PFS率改善相关，同时达到CR_V_和CR_H_的患者5年PFS率改善更为显著（95％对61％，*P*＝0.004）；auto-HSCT前应用诱导治疗还可减少移植并发症的发生率（9.4％对28.3％，*P*＝0.033）。移植前诱导治疗可以使患者更快地达到疾病缓解，改善患者的体能状态，从而使更多患者获得移植机会[18],[23]。表4为国际较大宗关于POEMS综合征患者进行auto-HSCT的报道，移植前进行诱导治疗的比例为38.9％～87％，诱导治疗方案包括沙利度胺、来那度胺、硼替佐米、美法仑及激素单药等，目前已有病例报道显示达雷妥尤单抗用于移植前的诱导治疗取得较好疗效。

专家组推荐进行auto-HSCT前予诱导治疗，诱导治疗尽可能使患者在移植前达到CR_H_，主要目的是改善患者病情，提高对移植的耐受力，降低移植相关并发症。在选择诱导治疗方案时需尽量避免对造血干细胞有蓄积毒性的药物，如含来那度胺方案的疗程数应≤4个，尽可能避免应用烷化剂如美法仑、环磷酰胺，以避免随后的造血干细胞动员采集失败和（或）造血重建延迟。在目前的新药时代，专家组推荐诱导治疗采用以新药为主的方案，为尽快达到血液学缓解，建议有条件者可加用CD38单抗。适合移植患者的诱导治疗可选择下述方案：①硼替佐米/地塞米松（Vd）±达雷妥尤单抗；②卡非佐米/地塞米松（Kd）±达雷妥尤单抗；③来那度胺/地塞米松（Rd）；④来那度胺/硼替佐米/地塞米松（RVd）；⑤硼替佐米/环磷酰胺/地塞米松（VCD）。考虑到性价比及疗效，建议一线治疗首选Rd方案或Vd方案。

（2）造血干细胞动员和预处理：目前报道的POEMS综合征患者的动员方案主要包括化疗（大剂量环磷酰胺或依托泊苷+粒细胞集落刺激因子（G-CSF）、G-CSF单药（占大多数）±趋化因子受体4（CXCR4）拮抗剂等。目前关于预处理的报道基本参考多发性骨髓瘤的预处理方案，尚无研究比较各预处理方案的优劣。

由于POEMS综合征患者克隆浆细胞水平低，且仅需做一次移植就可获得较好的疗效，专家组建议造血干细胞动员仅需采集单次移植的细胞数即可。因此，建议应用G-CSF 5～10 µg·kg^−1^·d^−1^按需联合CXCR4拮抗剂的方案。要求采集CD34^+^细胞数不少于2.0×10^6^/kg，理想采集量为5.0×10^6^/kg[24]。预处理采用美法仑200 mg/m^2^或140 mg/m^2^（根据肾功能或其他合并症进行调整）[18]。

（3）移植后巩固和维持治疗：目前尚无相关文献报道POEMS综合征患者行auto-HSCT后应用巩固或维持治疗。中山大学附属第一医院的经验显示，auto-HSCT后患者的血液学缓解、VEGF缓解均可保持长期CR状态。专家组推荐，除移植后疗效没有达到CR_H_和CR_V_的患者外，auto-HSCT后可以不进行巩固或维持化疗。

2. 不适合移植患者的治疗选择：目前无随机对照研究对比不适合移植的POEMS综合征患者应用不同方案的疗效。来那度胺目前已广泛用于POEMS综合征的治疗，北京协和医院的一项Ⅱ期临床试验纳入41例患者，Rd方案12个疗程的结果显示CR_H_率为46％，CR_V_率为43％[25]。另一项应用Rd方案治疗103例患者的回顾性研究也获得类似结果，CR_H_率为48％，CR_V_率为48％，CR_H_率或CR_V_率为66％，3年PFS率为65％，OS率为83％[20]。泊马度胺目前仅有个案报道。北京协和医院一项研究应用Vd方案（硼替佐米+地塞米松）治疗69例患者，CR_H_率为46％，CR_V_率为43.9％，临床/神经系统反应率为88％[21]。来自上海长征医院的一项研究采用VCD方案（硼替佐米+环磷酰胺+地塞米松）治疗20例患者，CR_H_率为41％，CR_V_率为76％，临床/神经系统反应率为95％[26]。也有个案报道显示卡非佐米和达雷妥尤单抗应用于新诊断不适合移植的患者[27]。

对于不适合移植的POEMS综合征患者，专家组推荐采用含新药的诱导治疗方案，上述适合移植的方案均可用于不进行移植的患者。如患者病情较轻，不伴有水肿、浆膜腔积液等，建议应用含来那度胺的方案。如病情危重，伴有水肿及多浆膜腔积液等，推荐加用达雷妥尤单抗、硼替佐米等药物。不适合移植的患者是否需要维持治疗尚存在争议，建议至少规律化疗8～9个疗程，然后进行巩固和维持治疗。

3. 复发患者的治疗：目前关于复发POEMS综合征的数据很少，auto-HSCT后复发患者更少。北京协和医院分析2004—2023年一线进行auto-HSCT后复发的100例患者，78例（78％）的患者接受Rd方案化疗，12例接受Vd方案，5例接受Td方案，3例接受Mel/Dex方案，1例接受Pd方案（泊马度胺+地塞米松）。进行血液学缓解程度评估的77例患者中29例（37.7％）达到CR_H_，分别有63例和10例患者达到CR_V_（VEGF<600 pg/ml，基线水平升高）和VEGF部分缓解（PR_V_）（基线VEGF>1 200 pg/ml时，VEGF至少降低50％）[28]。也有研究报道复发POEMS综合征患者应用挽救性第二次auto-HSCT[29]。美国克利夫兰诊所应用达雷妥尤单抗治疗7例复发POEMS综合征患者，其中5例可评估血液学疗效，总反应率（ORR）为100％，2例（40％）达到VEGF非常好的部分缓解（VGPR_V_），3例（60％）达到PR_V_；4例可评估VEGF疗效，2例（50％）达到CR_V_，2例（50％）达到PR_V_；临床反应评估1例（14％）达到临床完全缓解（CR_C_），5例（72％）达到PR_C_[30]。梅奥诊所应用以达雷妥尤单抗为基础的方案（包括联合来那度胺、泊马度胺、卡非佐米）治疗16例复发POEMS综合征，12例（75％）患者有效，其中9例（56％）达到CR/VGPR_H_，7例（44％）达到CR_V_，5例（31％）达到PET-CT完全缓解；在以卡非佐米为基础治疗的6例患者中，5例（83％）有反应，3例达到CR/VGPR_H_，1例达到PR_H_，1例有临床反应[31]。华中科技大学同济医学院附属同济医院报道了1例经来那度胺治疗后进展的POEMS综合征患者应用B细胞成熟抗原（BCMA）CAR-T细胞治疗取得较好的疗效[32]。

专家组建议根据患者既往一线治疗用药、疗效、药物相关不良反应及疗效持续时间、患者的体能状态和重要器官功能进行选择，可选择的方案有免疫调节剂（IMiD）（如来那度胺、泊马度胺）、蛋白酶体抑制剂（PI）（如硼替佐米、卡非佐米）、达雷妥尤单抗及上述药物的组合方案。

4. 支持治疗：对于合并内分泌病变患者可予胰岛素、甲状腺激素、性激素等替代治疗；对于水肿、多浆膜腔积液患者可予输注白蛋白、利尿、积液引流等改善症状；周围神经病变可予理疗及营养神经等治疗。部分患者可出现严重的心肺功能损害，需要多专科紧急干预，必要时可能需要重症监护支持。

六、疗效评价

POEMS综合征的疗效判断参照Humeniuk等[33]2013年在《Blood》上发表的标准，其中血液学疗效评价参照多发性骨髓瘤的疗效标准，见表5。

**表5:** POEMS综合征的疗效评价

疗效类型	疗效标准
血液学疗效（H）	CR_H_：骨髓恢复正常（如果基线阳性），血清和尿液免疫固定电泳阴性； VGPR_H_：M蛋白减少>90％（要求基线M蛋白≥5 g/L）； PR_H_：M蛋白减少>50％（要求基线M蛋白≥10 g/L）； NR_H_：未达PR_H_； PD：血清和（或）尿液中再次出现M蛋白，或较最低点增加>25％（要求M蛋白增加绝对值≥5 g/L）
VEGF疗效（V）	CR_V_：血清VEGF恢复正常； PR_V_：降低≥50％； NR_V_：未达PR_V_； PD：持续（≥2次）VEGF升高≥771 pg/ml或VEGF自治疗后最低点持续升高50％
影像学疗效（R）	CR_R_：FDG摄入恢复至基线水平； PR_R_：FDG摄入减少≥50％； NR_R_：FDG摄入减少<50％； PD：病灶SUVmax升高30％或新发浓聚灶
临床疗效	患者和医师对症状和体征的定性报告； 临床改善（Ci）； 临床进展（Cp）； 混合临床反应（Cm）； 临床稳定性（Cs）
神经疗效	由临床检查、神经生理学、改良Rankin评分和ONLS定义。进展定义为ONLS增加≥1分，功能恶化

**注** CR：完全缓解；VGPR：非常好的部分缓解；PR：部分缓解；NR：无应答；PD：疾病进展；VEGF：血管内皮生长因子；FDG：^18^F-氟代脱氧葡萄糖；ONLS：总体神经病变限制性评估量表；SUVmax：最大标准化摄取值

专家组建议POEMS综合征的疗效评价参考上述综合评价，主要包括：①临床疗效：评价神经病变、液体负荷、肺动脉高压等主要临床异常指标的改善情况；②血液学疗效：评价M蛋白的清除情况；③血清VEGF疗效：评价血清VEGF的下降情况。需注意，临床疗效评价是主观的，统一的应用标准是血液学和VEGF疗效。神经病变的疗效存在滞后情况，一般在治疗完成后6个月才有明显的改善，最大神经疗效需等待2～3年。其他临床表现如水肿、视乳头水肿、皮肤变化等通常会较快改善。最佳PET-CT反应也可能滞后6～12个月。
